# Effect of Nanometals and Pulsed Electric Field (PEF) on the Germination Capacity of Seeds and Antioxidative Properties of Seedlings of Sunflower

**DOI:** 10.3390/plants14162512

**Published:** 2025-08-12

**Authors:** Magdalena Kachel-Górecka, Karolina Sokal, Małgorzata Stryjecka

**Affiliations:** 1 Department of Machine Operation and Production Processes Management, Faculty of Production Engineering, University of Life Sciences in Lublin, 28 Głęboka St., 20-612 Lublin, Poland; karolina.sokal@up.lublin.pl; 2 Institute of Human Nutrition and Agriculture, The University College of Applied Sciences in Chełm, Pocztowa 54, 22-100 Chełm, Poland; mstryjecka@panschelm.edu.pl

**Keywords:** seedlings, sprouts, sunflowers, metal nanoparticles, PEF, antioxidative properties

## Abstract

The increasing integration of physical and nanotechnological treatments in agriculture has unlocked new possibilities for enhancing seed performance and the functional properties of seedlings. This study aimed to determine the effect of the coupled use of pulsed electric field (PEF) and the soaking (coating) of sunflower seeds in metal nanoparticles (AgNP and CuNP) on their germination capacity and on the stem and root length, content of pigments (chlorophyll *a*, chlorophyll *b*, carotenoids), color profile, and antioxidative properties (FRAP, polyphenols, TPC, ABTS, and DPPH) of sunflower seedlings. The study results enable the drawing of explicit conclusions that the higher PEF energy applied (5.5 kJ kg^−1^) and seed treatment with nanoparticle solutions, in most cases, diminished the germination capacity of sunflower seeds (from 3.50 to 44.11%) compared to the control samples. A decreased seedling stem length was determined at both PEF energy levels tested, i.e., 1 kJ kg^−1^ and 5.5 kJ kg^−1^, with the values obtained being 11.86% to 46.14% lower compared to the respective control samples. The root length of the seedlings decreased as well, i.e., by 7.34 to 41%. The content of chlorophyll *a* (chl *a*) increased in the seedlings from all experimental variants compared to the control, whereas that of chlorophyll *b* (chl *b*) decreased by 3.24 to 7.86% in the control variant with PEF and CuNP. The FRAP value, total content of polyphenols, and TPC ranged from 10.20 to 12.95 (mg TE g^−1^ DM), from 42.23 to 49.19 (mg GAE g^−1^ DM), and from 20.20 to 23.90 (mg GAE g^−1^ DM), respectively, and showed an upward trend compared to the control samples. The results of this study indicate that further research is needed to understand how the analyzed treatments affect seedling growth and demonstrate reduced germination capacity and enhanced antioxidant activity due to the synergistic effect of a high PEF and nanoparticle solutions.

## 1. Introduction

Sunflower (*Helianthus annuus* L.) is one of the most popular species of oil plants. Its seeds are commonly used as a snack; as an additive to salads, bakery products (breads), cosmetics, and greasing products; as a raw material for oil pressing; as a feedstuff for livestock; and as a biodiesel component [1,2]. Long-term and extensive research addressing this plant have enabled discovering its natural bioactive compounds, being of significant importance to pharmacotherapy and medical chemistry. Owing to these findings, sunflower seedlings were found utile in ethnomedicine for the treatment of multiple diseases, including cardiac diseases, diarrhea, hypertension, rheumatism, numerous infections of the bronchi, larynx, lungs, and cough [3,4]. Sunflower seeds and sprouts contain valuable components potent to prevent cardiovascular diseases and exhibiting anti-inflammatory, antidiabetic, antimicrobial, antihypertensive, and wound-healing properties. These effects are due to their phenolic compounds, flavonoids, carotenoids, polyunsaturated fatty acids, and vitamins [5,6]. Consumers are becoming more health conscious, which has led to a growing demand for sprouts, which can be eaten fresh or cooked, as appetizers or main dishes, and offer many health benefits while adding flavor to meals. In addition, they are well-suited for home or urban cultivation due to their minimal space requirements and rapid growth. Continuous temperature increases, drought, floods, and environmental contamination pose serious threats to plant seed germination in natural conditions, and these challenges are expected to become increasingly common worldwide.

Germination constitutes the initial phase of a seed’s physiological activity, culminating in seedling emergence. The seed shell serves a protective function to the embryo. Once it breaks, a sprouting root is the first tissue having an immediate contact with soil or water under natural growing conditions. According to the few available research works, the germination of sunflower seeds increases their nutritional value by increasing the content of phytochemicals [7,8,9]. The germination process is an indispensable stage of plant growth, when seeds leave the dormancy stage to produce seedlings. It can be initiated only when the critical moisture content has been achieved by, e.g., soaking the seeds in water as a pre-conditioning stage, in order to ensure their rapid germination and growth [10,11]. In addition, the metabolic adaptations that occur in seeds during germination enhance protein quality and other nutritional properties, making them promising candidates for healthy food products [12]. Plant seeds mainly act as a reservoir of valuable compounds for sprout development, whereas the germination process increases their total antiradical activity (40,202 μg Trolox g^−1^) compared to seeds (1456–25,991 μg Trolox g^−1^) [13]. Recent scientific studies have increasingly focused on identifying novel strategies to enhance and support this process. Pulsed electric field (PEF) technology and nanotechnology are regarded as innovative solutions for industrial-scale applications. The first is a non-thermal process involving the treatment of biological materials with short high-frequency impulses. To this end, a material sample is placed between electrodes to which an electric field is applied for very short periods of microseconds or milliseconds in order to induce cell membrane permeabilization [14,15]. When seed germination needs to be aided, the PEF parameters applied are expected to induce reversible cell permeabilization which does not result in nutrient leaching or any permanent damage, while preserving cell viability [16,17,18]. PEF helps to enhance water uptake with NP additives, stimulate the production of secondary metabolites, and improve the efficiency of germination [15].

According to Boussetta et al. [19], PEF treatments mainly result in the formation of micro- and nano-pores with low-temperature effects, which is particularly important in the case of heat-sensitive materials. However, as Bakhshabadi et al. [20] claimed, seed exposure to PEF might cause damage to oil cells and facilitate oil extraction without any temperature effects prior to the extraction. In turn, a study by Dannehl [21] proved that the effects of electricity (strong and weak electric fields, magnetic field, and electric current) on plant growth and selected plant metabolites can be classified as abiotic stress factors affecting plants.

Additionally, nanotechnology may seem a promising discipline in solving problems encountered during plant cultivation. It has recently become a prospective alternative offering a viable solution to the current problems through the use of materials in the nano-scale of 1 to 100 nm. Nanomaterials exhibit enhanced reactivity and a high surface-to-volume ratio, which makes them more efficient than their bulk counterparts. Implementation of nanotechnology to agriculture proves effective in the production of nanopesticides, nanoherbicides, and nanofertilizers, or the use of nanoparticles for the coating of seeds, which increases their protection against pests and improves their germination indices in various cropping systems [22,23]. Nanoparticles are synthesized using various methods, including chemical precipitation, thermal degradation, and sol–gel processes [24,25]. Apart from these methods, green synthesis has proved effective in delivering a considerable number of nanoparticles consisting of metals or metal oxides via biosynthesis pathways of algae, fungi, or plants [26].

The most popular nanomaterials used in agriculture include gold (Au), silver (Ag), copper (Cu) [27], magnesium oxide (MgO), calcium oxide (CaO), zinc oxide (ZnO), and titanium oxide (TiO_2_) [28,29]. A few research works have proved the NPs of heavy metals, like Fe, Ag, Zn, Mn, Cu, and Se, as well as metal oxides, like ZnO or Fe_3_O_4_, to be effective initiators of sprout growth [30,31,32]. However, available research works have not provided in-depth explanation of the interactions of the applied nanoparticles with plants. There have been several conflicting reports addressing nanoparticles regarding their accumulation, translocation, absorption, biotransformation, and toxicity in various plant species [33]. The majority of scientific works report adverse effects of AgNP and CuNP on seed germination and seedling growth, while showing no effect that would depend on the concentration of both nanoparticles and plant species. Botanical changes (either increase or decrease) triggered in response to AgNP exposure are observed in reactive oxygen species’ generation, superoxide dismutase activities, as well as the contents of total chlorophyll, carotenoids, and ascorbate, etc. These processes lead to abnormal morphological changes and the suppression of photosynthesis.

According to Prażak et al. [34], AgNPs increased seedling height, biomass, and net photosynthesis in two bean cultivars, ‘Bali’ and ‘Delfina’. The application of different doses of AgNPs to Nelumbo nucifera resulted in a significant increase in its biochemical parameters compared to the control group [35]. Another parameter of AgNPs, which affects the rate of their absorption by plant roots, is their electrokinetic potential (ζ-potential), generated near the NP surface by a layer of stabilizing molecules used to preserve their optimal stability [36]. Research by Abd El-Aziz et al. [37] showed that plant response to AgNPs was dose-dependent, resulting in either their growth enhancement or inhibition. Exposure to selected and sequentially applied AgNPs promoted plant growth more effectively than in control plants; however, both low and high concentrations adversely affected growth.

The response of rice (*Oryza sativa* L.) to CuONPs has been extensively studied using various NP concentrations and in various experimental systems. Most of these studies have demonstrated the growth inhibition of rice plants after exposure to CuO nanoparticles, which was mainly due to the oxidative stress generated [38].

However, as Arnott et al. [39] reported, the use of metal-based nanoparticles promoted plant germination and increased seedling vigor. In addition, most literature sources have reported on the equally-effective or enhanced germination and vigor growth upon seed nanoconditioning. Most findings from research entailing nanoconditioning techniques have been implemented in agricultural practice. The issue of the potential mechanisms underlying nanoparticle (NP) interactions with plant cells, such as oxidative stress and metabolic regulation, is a key research area that can significantly enhance our understanding of plant adaptive and defense strategies. Advances in electron microscopy (TEM) and analyses of antioxidative indices are enabling increasingly deeper insights into these complex processes. However, a lack of literature reports on the effects of the joint application of the PEF technique and nanoparticles on seeds has prompted us to undertake a study aimed to determine the effect of the coupled use of metal nanoparticles, including AgNP and CuNP, in two solutions (25 and 50 mL L^−1^), and PEF treatment at 1 and 5.5 kJ kg^−1^ on the germination capacity of sunflower seeds and antioxidative properties of grown seedlings. In order to extend their storage stability, the sunflower seedlings were dried, and the resulting material was analyzed for antioxidative properties, including total oxidative activity (FRAP), content of polyphenols, presence of phenolic compounds (TPC), antioxidative potential (ABTS), total antioxidative activity (DPPH), as well as the contents of carotenoids and chlorophyll *a* and *b*.

In view of the above, a research hypothesis was advanced that the coupled use of a pulsed electric field (PEF) with silver and copper nanoparticles in certain solutions (as specified above) and at various energy levels (as specified above) would positively affect the germination capacity of sunflower seeds and antioxidative properties of sunflower seedlings.

## 2. Results

### 2.1. Transmission Electron Microscopy (TEM) Analyses

**Figure 1:**
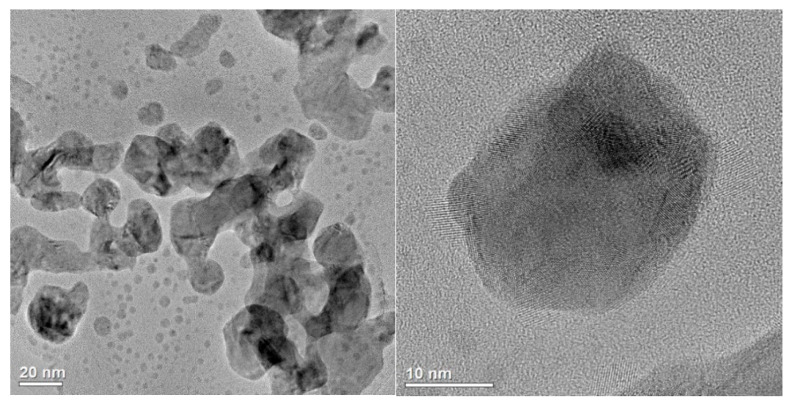
HR-TEM micrographs of the AgNP sample.

Analyses conducted by means of high-resolution transmission electron microscopy (HR-TEM) (Figure 1) demonstrated the presence of fine nanoparticles of Ag (AgNPs) with a spherical morphology and crystalline structure in the analyzed sample. They allowed establishing that AgNPs reached various sizes ranging from a few to several nanometers. In addition, crystallites coated with an amorphous (stabilizing) substance were observed locally.

**Figure 2:**
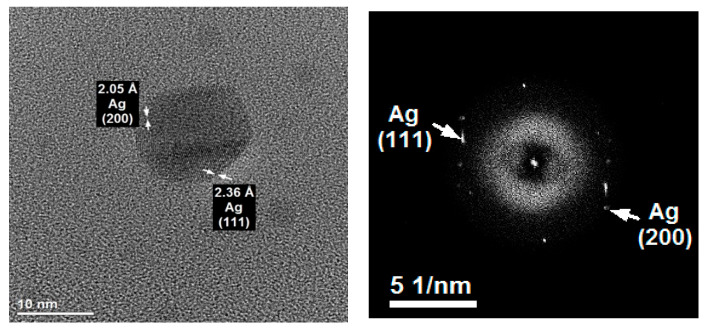
Phase identification in the AgNP sample using HR-/TEM and FFT. The arrows in the image represent the interplanar distances corresponding to the lattice planes (200 and 111).

The Ag crystallites occurred most often as single bodies and rarely as agglomerates of crystallites (Figure 2). The phase identification conducted in the AgNP sample based on HR-TEM and FFT (fast Fourier transform) imaging confirmed the presence of Ag crystallites in the metallic form based on the characteristic interplanar distances of 2.05 Å and 2.36 Å, corresponding to the (200) and (111) lattice planes.

**Figure 3:**
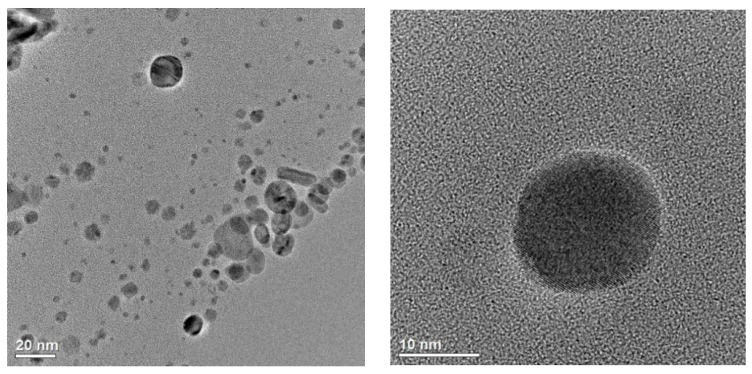
HR-TEM micrographs of the CuNP sample.

In addition, the TEM analyses (Figure 3) demonstrated the presence of fine nanoparticles of Cu (CuNPs), mainly with a spherical morphology and crystalline structure. The Cu nanoparticles were observed to reach various sizes ranging from a few to several nanometers. Locally, they mainly occurred as single crystallites coated with an amorphous (stabilizing) substance and also in agglomerates.

### 2.2. Evaluation of the Germination Capacity of Sunflower Seeds

The results of the sunflower seed germination capacity assessment conducted in the present study are collated in Table 1. The germination process was conducted under laboratory conditions. The germination capacity of the control seeds exposed to PEF at 1 kJ kg^−1^ reached 91.70% and of those treated at 5.5 kJ kg^−1^, 94.3%, being 0.77 and 3.63% higher, respectively, compared to the crude untreated control. The coupled use of metal nanoparticles with PEF in the variants with AgNPs and CuNPs applied at 25 mL L^−1^ and with a PEF of 1 kJ kg^−1^ resulted in a 96.30% germination capacity in the variant with AgNPs and 96.30% in the variant with CuNPs. Compared to the control variant with the same energy provided (1 kJ kg^−1^), the germination capacity determined for both NPs was higher by 5.02%. In the case of AgNP and CuNP application at 25 mL L^−1^ and a PEF at 5.5 kJ kg^−1^, the germination capacity of the seeds decreased significantly by 52.70% in the AgNP variant and by 71.70% in the CuNP variant, compared to the respective controls. The obtained values were lower by 23.97 and 44.11% compared to the control sample treated at a PEF of 5.5 kJ kg^−1^.

In the experimental variants with AgNPs and CuNPs applied in the solution of 50 mL L^−1^ and with a PEF energy of 1 kJ kg^−1^, the germination capacity of the seeds reached 93.30% and 89.70%, respectively (Table 1). In the case of the seeds treated with AgNPs, it was higher by 1.74%, and in the case of those treated with CuNPs, it was lower by 2.18%, compared to the respective control sample. In the variant with nanoparticles of both metals (Ag and Cu) applied at 50 mL L^−1^ with a PEF of 5.5 kJ kg^−1^, the germination capacity of the seeds decreased negligibly to 90.70% and 91.00%. The results obtained were lower by 3.82% and 3.50% compared to the respective control samples.

### 2.3. Evaluation of Stem Length and Root Length of Sunflower Seedlings

Table 1 also provides the results of measurements of the stem length of sunflower seedlings performed on day seven of their growth. The shoot lengths of the seedlings grown from the PEF-treated control seeds reached 57.70 mm (control 5.5 kJ kg^−1^) and 70.70 mm (control 1 kJ kg^−1^). The PEF treatment of seeds at 5.5 kJ kg^−1^ contributed to stem length shortening by 17.57%, compared to the crude control sample (70 mm). Soaking the seeds in the 25 mL L^−1^ AgNP solution followed by their PEF treatment at 1 kJ kg^−1^ caused the shoot length of seedlings to increase by 22.63%, compared to the respective control. With the same AgNP solution but a PEF energy of 5.5 kJ kg^−1^, seedling shoot length decreased by as much as 34.66%, compared to the control sample treated at 5.5 kJ kg^−1^. After seed soaking in a 50 mL L^−1^ AgNP solution and PEF treatment at 1 kJ kg^−1^ and 5.5 kJ kg^−1^, the shoot length increased by 11.32% in the 1 kJ kg^−1^ variant and by as much as 46.79% in the 5.5 kJ kg^−1^ variant.

Seed soaking in 25 and 50 mL L^−1^ solutions of CuNPs followed by PEF treatment at 1 kJ kg^−1^ contributed to stem length decrease by 26.03% and 35.36%, respectively, compared to the respective control sample. For both CuNP solutions and a PEF energy of 5.5 kJ kg^−1^, the shoot length of the seedlings increased by 6.9% and 4.51%, compared to the PEF-treated control. In turn, the differences noted between all experimental variants with CuNPs and the PEF-treated control were lower by 11.86 (CuNP 25 mL L^−1^ and 5.5 kJ kg^−1^) and 25.29% (CuNP 25 mL L^−1^ and 1 kJ kg^−1^).

The results of the measurements of sunflower seedling root length on day seven of the experiment are presented in Table 1. In the control PEF-treated samples, the root lengths of the seedlings reached 60.30 mm (control 1 kJ kg^−1^) and 76.30 mm (control 5.5 kJ kg^−1^). The PEF treatments at the energy levels tested also contributed to root length increases by 2.20% and 29.32%, respectively, compared to the crude control sample (59 mm).

After seed soaking in the 25 and 50 mL L^−1^ solutions of AgNPs and their PEF treatment at 1 kJ kg^−1^, the root lengths of the seedlings increased by 33.83 and 32.15%, compared to the respective control sample. Similar differences were noted between the above samples and the crude control not exposed to PEF. After PEF use at 5.5 kJ kg^−1^, the root length decreased only in the variant with AgNPs applied at 25 mL L^−1^ and was lower by 7.34% compared to the respective control sample. In addition, sunflower seedling root length was greater compared to the PEF-treated control sample by 19.83% in the variants with AgNP 25 mL L^−1^ and 5.5 kJ kg^−1^ and by 35.08% in the variants with AgNP 50 mL L^−1^ and 5.5 kJ kg^−1^. In the case of the seeds soaked in the 25 and 50 mL L^−1^ solutions of CuNPs and exposed to a PEF at 1 kJ kg^−1^, the root length of the seedlings increased slightly by 1.16% in the variant with the lower NP solution and decreased by 35.36% in the variant with the higher NP solution tested, compared to respective controls. In the variant with 25 mL L^−1^ CuNPs and a PEF energy of 5.5 kJ kg^−1^, the root length decreased significantly by 41.00% compared to the control PEF-treated sample and by 23.73% compared to the crude control. In turn, in the variant with a 50 mL L^−1^ CuNP solution and PEF energy of 5.5 kJ kg^−1^, the root length of the seedlings increased negligibly (by 0.52%) compared to the control PEF-treated sample and significantly (by 30.00%) compared to the crude control.

### 2.4. Seedling Color

Table 2 presents the results of color measurements of dried sunflower seedlings. The color parameters were determined in the CIEL*a*b* system, where the *L* parameter indicates relative sample brightness.

The color analysis of sunflower seedlings demonstrated they were relatively bright, with the *L* values ranging from 47.70 in the sample treated with a PEF energy of 1 kJ kg^−1^ and 25 mL L^−1^ CuNP solution to 55.09 in the sample treated with a PEF energy of 5.5 kJ kg^−1^ and 50 mL L^−1^ AgNP solution. The *L* color coordinate is a relative parameter indicative of the mixing of the primary color with a black color (low *L*) or white color (high *L*). In the case of the control samples treated with a PEF energy of 1 kJ kg^−1^ and 5.5 kJ kg^−1^, the *L* values decreased by 2.73% and increased by 7.28%, respectively, compared to the crude control sample not exposed to PEF.

The analysis of the study results showed a decrease in the discussed parameter by 4.89% (1 kJ kg^−1^ Cu25), 10.91% (5.5 kJ kg^−1^ Cu25), and 4.29% (1 kJ kg^−1^ Ag50), as well as its increase by 5.32% (1 kJ kg^−1^ Cu50), 2.01% (5.5 kJ kg^−1^ Cu50), 6.10% (1 kJ kg^−1^ Ag25), 6.26% (5.5 kJ kg^−1^ Ag25), and 9.85% (5.5 kJ kg^−1^ Ag50), compared to the seedlings from the crude control seeds not exposed to PEF.

After seed soaking in the AgNP solution, the *L* parameter values of the seedlings ranged from 48.00 (1 kJ kg^−1^ and 50 mL L^−1^ nanoparticles) to 55.09 (5.5 kJ kg^−1^ and 50 mL L^−1^ nanoparticles). The lower PEF energy applied (1 kJ kg^−1^) with 25 and 50 mL·L^−1^ solutions of the nanoparticles contributed to an increase in the *L* value by 9.08% and its decrease by 1.60%, compared to the control sample exposed to a PEF energy of 1 kJ kg^−1^. In turn, seed treatment with the higher PEF energy (5.5 kJ kg^−1^) and nanoparticle solutions of 25 and 50 mL L^−1^ caused a negligible (0.95%) decrease of this parameter and its increase by 2.40% compared to the seedlings grown from the control seeds exposed to a PEF energy of 5.5 kJ kg^−1^.

After sunflower seed soaking in the CuNP solution, the values of the *L* parameter determined in the seedlings ranged from 44.68 (5.5 kJ kg^−1^ and 25 mL L^−1^ nanoparticles) to 52.82 (1 kJ kg^−1^ and 50 mL L^−1^ nanoparticles). The lower PEF energy applied (1 kJ kg^−1^) with 25 and 50 mL L^−1^ solutions of the nanoparticles contributed a decrease in the discussed parameter by 2.21% and its increase by 8.28%, compared to the control sample exposed to a PEF energy of 1 kJ kg^−1^. In turn, seed treatment with the higher PEF energy (5.5 kJ kg^−1^) and CuNP solutions of 25 and 50 mL L^−1^ caused *L* values to decrease by 16.95% and 4.91%, respectively, compared to the 5.5 kJ kg^−1^ control sample.

The *a* and *b* color coordinates denote sample color. Negative *a* values indicate the contribution of the green color, whereas the positive ones indicate the contribution of the red color in the total color profile. The analysis of the measurements of parameters *a* and *b* demonstrated negative *a* values ranging from −4.25 in the sample exposed to PEF energy of 5.5 kJ kg^−1^ and 25 mL·L^−1^ CuNP solution to −1.28 in the control sample, pointing to the contribution of the green color in the total color profile of all sunflower seedling samples. It also showed increased values of the *a* parameter in the samples from all variants, compared to the crude control not exposed to PEF. The lower *a* values are indicative of the greater contribution of the green color in the total color profile.

After seed soaking in the AgNP solution, the values of the *a* parameter of the seedlings ranged from −3.96 (5.5 kJ kg^−1^ and 25 mL L^−1^ nanoparticles) to −1.44 (1 kJ kg^−1^ and 50 mL L^−1^ nanoparticles). The coupled use of the lower PEF energy (1 kJ kg^−1^) with 25 and 50 mL L^−1^ nanoparticle solutions caused the *a* value to decrease by 17.54% and 31.75%, respectively, compared to the respective control sample exposed to PEF. In turn, seed treatment with the higher PEF energy (5.5 kJ kg^−1^) and 25 and 50 mL L^−1^ nanoparticles contributed to the *a* value increasing by 110.64% and its decrease by 15.43%, compared to the control sample exposed to a PEF at 5.5 kJ kg^−1^.

After seed treatment with the CuNP solution, the values of the *a* parameter ranged from −4.25 (5.5 kJ kg^−1^ and 25 mL L^−1^ nanoparticles) to −2.94 (1 kJ kg^−1^ and 50 mL L^−1^ nanoparticles). The coupled use of the lower PEF energy (1 kJ kg^−1^) with 25 and 50 mL L^−1^ nanoparticle solutions caused the *a* value to increase by 60.19% and 39.34%, respectively, compared to the control sample exposed to a PEF at 1 kJ kg^−1^.

In turn, seed treatment at the higher PEF energy (5.5 kJ kg^−1^) and 25 and 50 mL L^−1^ solutions of the nanoparticles contributed to the *a* value increasing by 126.06% and its decrease by 7.45% compared to the control sample exposed to PEF at 5.5 kJ kg^−1^.

In the case of color parameter *b*, its negative values indicate the contribution of the green color and positive ones—of the yellow color in the total color profile. The analysis of *b* values demonstrated the contribution of the yellow color in the total color profile of all samples of sunflower seedlings. In addition, it showed *b* values to increase, compared to the control sample not exposed to PEF energy.

After sunflower seed soaking in the AgNP solution, the values of the *b* parameter determined in seedlings ranged from 22.73 (1 kJ kg^−1^ and 25 mL L^−1^ nanoparticles) to 27.56 (5.5 kJ kg^−1^ and 25 mL L^−1^ nanoparticles). The use of the lower PEF energy (1 kJ kg^−1^) with 25 and 50 mL L^−1^ nanoparticle solutions caused the *b* value to decrease by 12.00% and 11.07%, respectively, compared to the control sample exposed to PEF at 1 kJ kg^−1^. In contrast, the higher PEF energy applied (5.5 kJ kg^−1^) with 25 and 50 mL L^−1^ solutions of the nanoparticles contributed to the *b* value increasing by 9.28% and its decrease by 5.95%, respectively, compared to the control sample exposed to PEF at 5.5 kJ kg^−1^.

After sunflower seed soaking in the CuNP solution, the values of the *b* parameter fitted within the range from 22.31 (5.5 kJ kg^−1^ and 50 mL L^−1^ nanoparticles) to 27.34 (5.5 kJ kg^−1^ and 25 mL L^−1^ nanoparticles). The coupled use of the lower PEF energy (1 kJ kg^−1^) with 25 and 50 mL L^−1^ nanoparticle solutions contributed to their 4.14% and 1.20% decrease, respectively, compared to the control sample exposed to a PEF at 1 kJ kg^−1^. In turn, the higher PEF energy applied (5.5 kJ kg^−1^) at 25 and 50 mL L^−1^ solutions of the nanoparticles resulted in the *b* value increasing by 8.41% and its decrease by 11.54%, respectively, compared to the control sample exposed to PEF at 5.5 kJ kg^−1^.

The analysis of the values of parameter *C* showed that color chromaticity increased in all samples compared to the control sample not exposed to PEF.

After sunflower seed soaking in the AgNP solution, the *C* parameter values determined in the seedlings ranged from 22.8 (1 kJ kg^−1^ and 25 mL L^−1^ nanoparticles) to 27.85 (5.5 kJ kg^−1^ and 25 mL L^−1^ nanoparticles). The coupled use of the lower PEF energy (1 kJ kg^−1^) with 25 and 50 mL L^−1^ nanoparticle solutions caused a decrease in *C* values by 12.04% and 11.23%, respectively, compared to the control sample exposed to PEF at 1 kJ kg^−1^. In turn, the higher energy applied (5.5 kJ kg^−1^) with 25 and 50 mL L^−1^ solutions of the nanoparticles contributed to chromaticity increasing by 10.12% and its decrease by 5.97%, respectively, compared to the control sample exposed to PEF at 5.5 kJ kg^−1^.

The soaking of sunflower seeds in the CuNP solution resulted in *C* parameter values ranging from 22.38 (5.5 kJ kg^−1^ and 50 mL L^−1^ nanoparticles) to 27.67 (5.5 kJ kg^−1^ and 25 mL L^−1^ nanoparticles). The lower PEF energy (1 kJ kg^−1^) applied with 25 and 50 mL L^−1^ solutions of the nanoparticles contributed to chromaticity decreasing by 3.59% and 0.89%, respectively, compared to the control sample exposed to PEF at 1 kJ kg^−1^. In contrast, seeds’ exposure to the higher PEF energy (5.5 kJ kg^−1^) and nanoparticles at 25 and 50 mL L^−1^ resulted in *C* increasing by 9.41% and its decrease by 11.51%, respectively, compared to the seedlings from the control seeds exposed to PEF at 5.5 kJ kg^−1^.

The value of parameter *h* denotes the hue of the material’s yellow color. In all samples, it decreased compared to the control variant.

After sunflower seed soaking in the AgNP solution, the *h* values ranged from 81.82 (5.5 kJ kg^−1^ and 25 mL L^−1^ nanoparticles) to 86.42 (1 kJ kg^−1^ and 50 mL L^−1^ nanoparticles). Seed treatment with the lower PEF energy (1 kJ kg^−1^) and 25 and 50 mL L^−1^ nanoparticle solutions caused a slight increase in the value of this color parameter by 0.34% and 1.27%, respectively, compared to the control sample exposed to PEF at 1 kJ kg^−1^. In contrast, the higher PEF energy applied (5.5 kJ kg^−1^) with 25 and 50 mL L^−1^ solutions of the nanoparticles decreased the *h* value by 4.57% and increased it by 0.52%, respectively, compared to the control sample exposed to PEF at 5.5 kJ kg^−1^.

After sunflower seed soaking in the CuNP solution, the values of parameter *h* fitted within the range from 81.18 (5.5 kJ kg^−1^ and 25 mL L^−1^ nanoparticles) to 85.55 (5.5 kJ kg^−1^ and 50 mL L^−1^ nanoparticles).

Seed treatment with the lower PEF energy (1 kJ kg^−1^) and 25 and 50 mL L^−1^ solutions of the nanoparticles contributed to the hue angle decreasing by 3.66% and 2.23%, respectively, compared to the control sample exposed to a PEF at 1 kJ kg^−1^. In turn, the higher PEF energy (5.5 kJ kg^−1^) and 25 and 50 mL L^−1^ nanoparticle solutions resulted in the *h* value decreasing by 5.32% and 0.22%, respectively, compared to the control sample exposed to PEF at 5.5 kJ kg^−1^.

Table 3 presents the contents of carotenoids and chlorophyll *a* and *b* in sunflower seedlings after seeds’ exposure to a pulsed electric field (1 kJ kg^−1^ and 5.5 kJ kg^−1^) and soaking in solutions of silver and copper nanoparticles (25 and 50 mL L^−1^).

The analysis of the chlorophyll *a* content of sunflower seedlings demonstrated its values as being between 36.4 in the control samples not exposed to PEF energy and 41.6 mg 100 g^−1^ DM in the sample treated with a PEF energy of 5.5 kJ kg^−1^ and 50 mL L^−1^ AgNP solution. The content of chlorophyll *a* increased in the seedlings from all experimental variants compared to the control, with the greatest increase noted for the variant with a PEF energy of 5.5 kJ kg^−1^ and 50 mL L^−1^ solution of silver nanoparticles (14.29%).

After sunflower seed soaking in the AgNP solution, the chlorophyll *a* content of the seedlings ranged from 38.2 (1 kJ kg^−1^ and 25 mL L^−1^ nanoparticles) to 41.6 mg 100 g^−1^ DM (5.5 kJ kg^−1^ and 50 mL L^−1^ nanoparticles). Seed exposure to the lower PEF energy (1 kJ kg^−1^) and 25 and 50 mL L^−1^ solutions of the nanoparticles resulted in increased values of the discussed parameter of 4.09% in the 25 mL L^−1^ variant and 5.99% in the 50 mL L^−1^ variant compared to the control sample exposed to PEF at 1 kJ kg^−1^. In turn, the higher energy applied (5.5 kJ kg^−1^) with 25 and 50 mL L^−1^ nanoparticle solutions increased the chlorophyll *a* content of the seedlings by 9.24% and 13.04%, respectively, compared to the control sample exposed to a PEF at 5.5 kJ kg^−1^.

After sunflower seed soaking in the CuNP solution, the chlorophyll *a* content of the seedlings fitted within the range from 38.4 (1 and 5.5 kJ kg^−1^ and 25; 50 mL L^−1^ nanoparticles) to 38.7 mg 100 g DM^−1^ (5.5 kJ kg^−1^ and 25 mL L^−1^ nanoparticles). The higher PEF energy applied (1 kJ kg^−1^) with 25 and 50 mL L^−1^ solutions of the nanoparticles contributed to its increase by 4.63% at both NP solutions compared to the control sample exposed to a PEF at 1 kJ kg^−1^. In turn, the higher PEF energy (5.5 kJ kg^−1^) applied with 25 and 50 mL L^−1^ nanoparticle solutions caused a minimal increase in chlorophyll *a* content, i.e., by 5.16% and 4.08%, respectively, compared to the control sample exposed to a PEF at 5.5 kJ kg^−1^.

The analysis of the chlorophyll *b* content of sunflower seedlings demonstrated its values as ranging from 16.4 in the seedlings from the seeds exposed to a PEF energy of 5.5 kJ kg^−1^ and the 25 mL L^−1^ solution of copper nanoparticles to 22.8 mg 100 g^−1^ DM in the seedlings from seeds exposed to a PEF energy of 5.5 kJ kg^−1^ and the 25 mL L^−1^ solution of silver nanoparticles. It also showed an increase in its content in the following samples compared to the crude control sample not exposed to PEF. Seed treatment with a PEF energy of 1 kJ kg^−1^ and copper nanoparticles at 25 mL L^−1^ caused a 6.77% decrease in chlorophyll *b* content, whereas a PEF energy of 5.5 kJ kg^−1^ and 25 mL L^−1^ solution of copper nanoparticles caused a 14.58% increase in its content compared to the control.

After sunflower seed soaking in the AgNP solution, the content of chlorophyll *b* determined in the seedlings ranged from 20.7 (1 kJ kg^−1^ and 50 mL L^−1^ nanoparticles) to 22.8 mg 100 g^−1^ DM (5.5 kJ kg^−1^ and 25 mL L^−1^ nanoparticles). Seed treatment with the lower PEF energy (1 kJ kg^−1^) and soaking in 25 and 50 mL L^−1^ solutions of the nanoparticles increased its values by 15.14% in the 25 mL L^−1^ variant and 11.89% in the 50 mL L^−1^ variant compared to the control sample exposed to a PEF at 1 kJ kg^−1^. In turn, seed exposure to the higher PEF energy (5.5 kJ kg^−1^) and 25 and 50 mL L^−1^ solutions of the nanoparticles increased the chlorophyll *b* content of the seedlings by 28.09% and 24.72%, respectively, compared to the control sample exposed to a PEF at 5.5 kJ kg^−1^.

After sunflower seed soaking in the CuNP solution, the content of chlorophyll *b* analyzed in the seedlings fitted within the range from 17.9 (1 kJ kg^−1^ and 25 mL L^−1^ nanoparticles) to 20.6 mg 100 g^−1^ DM (5.5 kJ kg^−1^ and 50 mL L^−1^ nanoparticles). The application of the lower PEF energy (1 kJ kg^−1^) and 25 and 50 mL L^−1^ nanoparticle solutions decreased its value by 3.34% and increased it by 7.03%, respectively, compared to the control sample exposed to PEF at 1 kJ kg^−1^. In turn, the higher PEF energy applied (5.5 kJ kg^−1^) and seed soaking in 25 and 50 mL L^−1^ solutions of the nanoparticles caused its decrease by 7.87% and increase by 15.73%, respectively, compared to the control sample exposed to a PEF at 5.5 kJ kg^−1^.

The analysis of the total content of carotenoids in sunflower seedlings demonstrated its values to be within a range from 34.01 in the control sample exposed to a PEF at 1 kJ kg^−1^ to 39.4 mg 100 g^−1^ DM upon seed exposure to a PEF at 5.5 kJ kg^−1^ and 50 mL L^−1^ silver nanoparticle solution. The seedlings from the control sample treated with a PEF at 1 kJ kg^−1^ had a minimally lower content of carotenoids (by −1.45%), whereas those from the variant with a PEF at 5.5 kJ kg^−1^ showed a 2.02% increase in their content compared to the crude control sample not exposed to a PEF. All samples had a higher total carotenoid content compared to the crude control not exposed to PEF energy.

After sunflower seed soaking in the AgNP solution, the total content of carotenoids in the seedlings ranged from 37.5 (1 kJ kg^−1^ and 25 mL L^−1^ nanoparticles) to 39.4 mg 100 g^−1^ DM (5.5 kJ kg^−1^ and 50 mL L^−1^ nanoparticles). Seed exposure to the lower PEF energy (1 kJ kg^−1^) and soaking in 25 and 50 mL L^−1^ nanoparticle solutions increased it by 15.14% in the 25 mL L^−1^ variant and 11.89% in the 50 mL L^−1^ variant, compared to the control sample exposed to a PEF at 1 kJ kg^−1^. In turn, the higher PEF energy applied (5.5 kJ kg^−1^) and seed soaking in 25 and 50 mL L^−1^ solutions of the nanoparticles increased it by 28.09% and 24.72%, respectively, compared to the control sample exposed to a PEF at 5.5 kJ kg^−1^.

After sunflower seed soaking in the CuNP solution, the total content of carotenoids ranged from 36.2 (1 kJ kg^−1^ and 25 mL L^−1^ nanoparticles) to 37.0 mg 100 g^−1^ DM (5.5 kJ kg^−1^ and 25 mL L^−1^ nanoparticles). When the seeds were exposed to the lower PEF energy (1 kJ kg^−1^) and soaked in 25 and 50 mL L^−1^ solutions of the nanoparticles, the grown seedlings had 7.33% and 6.16%, respectively, higher total carotenoid contents compared to the control sample exposed to a PEF at 1 kJ kg^−1^. In turn, the higher PEF energy (5.5 kJ kg^−1^) and 25 and 50 mL L^−1^ solutions of nanoparticles caused the value of this parameter to increase by 4.82% and 2.83%, respectively, compared to the control variant with a PEF of 5.5 kJ kg^−1^.

### 2.5. Antioxidative Potential

Table 4 provides results of the determinations of the total oxidative activity, content of polyphenols, content of phenolic compounds, and antioxidative activity against ABTS and DPPH radicals in sunflower seedlings.

In the present study, the total oxidative activity (FRAP) ranged from 10.20 for the crude control sample to 12.95 mg TE g^−1^·DM for the seedlings germinated from seeds soaked with a 25 mL L^−1^ CuNP solution and exposed to a PEF energy of 5.5 kJ kg^−1^. In all experimental variants, the application of a PEF and seed soaking in nanoparticles (AgNP, CuNP) increased the total oxidative activity of the analyzed sunflower seedlings. The exposure of the control seeds to a PEF (1 kJ kg^−1^ and 5.5 kJ kg^−1^) increased the FRAP value in both variants, i.e., by 8.63 and 6.99%, respectively, compared to the crude control. The greatest increase in the total oxidative activity was noted for the seedlings of seeds exposed to both energy levels (1 and 5.5 kJ kg^−1^) and soaked in both analyzed solutions of CuNP nanoparticles (25 and 50 mL L^−1^) as well as for the seedlings germinated from seeds exposed to a PEF energy of 1 kJ kg^−1^ and soaked in the 25 mL L^−1^ AgNP solution. The values obtained in these variants were higher than those determined for the respective control samples by 15.97% (1 kJ kg^−1^ CuNP25), 9.91% (1 kJ kg^−1^ Cu50 and Ag25), 18.69% (5.5 kJ kg^−1^ Cu25), and 13.14% (5.5 kJ kg^−1^ Cu50).

The content of polyphenols in the analyzed sunflower seedlings (Table 4) ranged from 42.23 for the control sample without seed exposure to a PEF to 49.19 mg GAE g^−1^ DW for the seedlings from seeds soaked in the 25 mL L^−1^ CuNP solution and exposed to a PEF energy of 5.5 kJ kg^−1^. The polyphenol content of the seedlings germinated from the control seeds treated with a PEF at both 1 and 5.5 kJ kg^−1^ was 3.23 and 4.82% higher, respectively, compared to the seedlings from the crude control variant (without PEF treatment).

The content of polyphenols increased in the seedlings from all experimental variants compared to the respective control samples. The greatest increase was noted for the seedlings from the seeds exposed to a PEF energy of 1 kJ kg^−1^ and 5.5 kJ kg^−1^ and soaked in both analyzed solutions of CuNP (25 and 50 mL L^−1^).

The results obtained in the present study for the TPC of sunflower seedlings ranged from 20.2 for those from the control seeds exposed to a PEF energy of 1 kJ kg^−1^ to 23.9 mg GAE g^−1^·DM for the seedlings germinated from the seeds exposed to a PEF at 5.5 kJ kg^−1^ and soaked in the 50 mL L^−1^ AgNP solution. A decrease in the content of phenolic compounds compared to the control not exposed to a PEF was observed for the seedlings from the control seeds treated with a PEF energy of 1 and 5.5 kJ kg^−1^. The values obtained for these variants were lower by 4.27% and 1.90%, respectively. A minimal decrease (by 1.42%) was noted in the seedlings from the seeds exposed to a PEF energy of 1 kJ kg^−1^ and soaked in the 25 mL L^−1^ AgNP solution.

After sunflower seed soaking in the AgNP solution, the total content of phenolic compounds (TPC) of the seedlings ranged from 20.8 (1 kJ kg^−1^ and 25 mL L^−1^ nanoparticles) to 23.9 mg GAE g^−1^·DM (5.5 kJ kg^−1^ and 50 mL L^−1^ nanoparticles). Seed exposure to the lower PEF energy (1 kJ kg^−1^) and their soaking in 25 and 50 mL L^−1^ nanoparticle solutions resulted in increased TPC values in the seedlings by 2.97% in the 25 mL L^−1^ variant and 12.38% in the 50 mL L^−1^ variant compared to the control sample exposed to a PEF at 1 kJ kg^−1^. In turn, the higher PEF energy applied (5.5 kJ kg^−1^) and seed soaking in 25 and 50 mL L^−1^ nanoparticle solutions caused the TPC to increase by 10.63% and 15.46%, respectively, compared to the control sample exposed to a PEF at 5.5 kJ kg^−1^.

After sunflower seed soaking in the CuNP solution, the TPC of the seedlings ranged from 21.5 (5.5 kJ kg^−1^ and 50 mL L^−1^ nanoparticles) to 22.6 mg GAE g^−1^ DM (5.5 kJ kg^−1^ and 25 mL L^−1^ nanoparticles). The application of the lower PEF energy (1 kJ kg^−1^) and 25 and 50 mL L^−1^ solutions of the nanoparticles contributed to TPC increasing by 6.93% and 7.43%, respectively, compared to the control sample exposed to a PEF at 1 kJ kg^−1^. In turn, seed exposure to the higher PEF energy (5.5 kJ kg^−1^) and 25 and 50 mL L^−1^ nanoparticle solutions caused TPC to increase by 9.18% and 3.86%, respectively.

The analysis of the antioxidative activity against the ABTS radical in sunflower seedlings showed its values to be between 78.2 in the control sample without PEF treatment and 84.7 μmol Trolox g^−1^ DM in the variant with a PEF of 5.5 kJ kg^−1^ and the 25 mL L^−1^ AgNP solution. An increase in the antioxidative activity compared to the control not exposed to PEF was observed in the seedlings germinated from the control seeds treated with a PEF of 1 and 5.5 kJ kg^−1^ (increase by 1.79% and 3.07%, respectively).

After sunflower seed soaking in the AgNP solution, the ABTS radical scavenging activity of the seedlings ranged from 80.6 (1 kJ kg^−1^ and 25 mL L^−1^ nanoparticles) to 84.7 μmol Trolox g^−1^ DM (5.5 kJ kg^−1^ and 25 mL L^−1^ nanoparticles). Seed exposure to the lower PEF energy (1 kJ kg^−1^) and their soaking in 25 and 50 mL L^−1^ nanoparticle solutions increased the value of the discussed parameter by 1.26% in the 25 mL L^−1^ variant and 3.77% in the 50 mL L^−1^ variant, compared to the control sample exposed to a PEF at 1 kJ kg^−1^. In turn, the higher PEF energy applied (5.5 kJ kg^−1^) and 25 and 50 mL L^−1^ solutions of nanoparticles caused its value to increase by 5.09% and 1.74%, compared to the seedlings germinated from the control seeds exposed to a PEF at 5.5 kJ kg^−1^.

After sunflower seed soaking in the CuNP solution, the antioxidative capability of seedlings determined in the ABTS assay ranged from 80.0 (5.5 kJ kg^−1^ and 25 mL L^−1^ nanoparticles) to 82.7 μmol Trolox g^−1^ DM (5.5 kJ kg^−1^ and 50 mL L^−1^ nanoparticles). Seed treatment with the lower PEF energy (1 kJ kg^−1^) and nanoparticle solutions of 25 and 50 mL L^−1^ contributed to its value increasing by 3.27% and 2.39%, respectively, compared to the seedlings from the control seeds exposed to a PEF at 1 kJ kg^−1^. In contrast, the higher PEF energy (5.5 kJ kg^−1^) applied together with seed soaking in 25 and 50 mL L^−1^ nanoparticle solutions caused a minimal decrease in the antioxidative capability of seedlings by 0.74% and its increase by 2.61%, compared to the control sample exposed to a PEF at 5.5 kJ kg^−1^.

The analysis of the total antioxidative activity against the DPPH radical in the sunflower seedlings ranged from 46.1 in those from the control variant with a PEF of 1 kJ kg^−1^ to 51.4 μmol Trolox g^−1^ DM in those from the variant with a PEF of 5.5 kJ kg^−1^ and 25 mL L^−1^ silver nanoparticle solution. The antioxidative activity of the seedlings germinated from the control seeds exposed to a PEF of 1 kJ kg^−1^ decreased minimally (by 1.28%), compared to those from the control seeds not exposed to a PEF, as well as increased (by 3.43%) in the variant with seed exposure to a PEF of 5.5 kJ kg^−1^.

After sunflower seed soaking in the AgNP solution, the total DPPH antioxidative activity ranged from 48.9 (1 kJ kg^−1^ and 50 mL L^−1^ nanoparticles) to 51.4 μmol Trolox g^−1^ DM (5.5 kJ kg^−1^ and 25 mL L^−1^ nanoparticles). Seed exposure to the lower PEF energy (1 kJ·kg^−1^) and their soaking in 25 and 50 mL L^−1^ nanoparticle solutions resulted in the activity increasing in the seedlings by 9.33% in the 25 mL L^−1^ variant and 6.07% in the 50 mL L^−1^ variant, compared to the control variant with seed exposure to a PEF at 1 kJ kg^−1^. In turn, the higher PEF energy applied (5.5 kJ kg^−1^) and seed treatment with 25 and 50 mL L^−1^ solutions of AgNPs caused this activity to increase in the seedlings by 6.42% and 6.00%, respectively, compared to the control variant with seed exposure to a PEF at 5.5 kJ kg^−1^.

After sunflower seed soaking in the CuNP solution, the total DPPH antioxidative activity fitted within the range from 48.4 (1 kJ kg^−1^ and 50 mL L^−1^ nanoparticles) to 49.3 μmol Trolox g^−1^ DM (5.5 kJ kg^−1^ and 50 mL L^−1^ nanoparticles). The application of the lower PEF energy (1 kJ kg^−1^) and seed soaking in 25 and 50 mL L^−1^ solutions of nanoparticles increased the antioxidative activity of seedlings by 5.86% and 4.99%, respectively, compared to the control seedlings germinated from seeds exposed to a PEF at 1 kJ kg^−1^. In turn, seed exposure to the higher PEF energy (5.5 kJ kg^−1^) and 25 and 50 mL L^−1^ solutions of nanoparticles increased the value of this parameter by 3.11% and 2.07%, respectively, compared to the control variant with seeds exposed to a PEF at 5.5 kJ kg^−1^.

## 3. Discussion

### 3.1. Effect of PEF and NPs on Germination and Morphological Growth

The most commonly used indices for assessing the impact of various factors on plant growth include: the seed germination percentage, root and stem length, and contents of total and dry matter of roots [40]. Undoubtedly, NP application may exert both positive and negative effect on the morphometric parameters of plants depending on such factors as: plant species and experimental conditions including the concentration and physicochemical parameters of nanoparticles, like their size and surface stabilizer used [40]. The seed dressing stage may contribute to the binding of the dressing agent to the seed coat, aiding water absorption by seeds and thereby promoting starch metabolism and seed germination. In addition, it may increase soluble sugar content, which also promotes seedling growth [41]. According to Cvjetko et al. [42], the plant root surface conveys a negative charge, whereas the addition of positively charged metal nanoparticles (AgNPs) improves their adhesiveness to parts of the plant, which ultimately leads to faster NP accumulation in plant tissues. Noteworthy in this case is also the impact of the physicochemical properties of nanoparticles, such as their size (it increases along with NP size decrease) [43], and type of stabilizer used [44]. In turn, Pandey et al. [45], who applied AgNP and AgNO_3_ in solutions of 100, 500 and 1000 mL L^−1^ to Indian mustard seeds, showed that AgNP application contributed to only a slight improvement in the root and stem length of seedlings. Furthermore, Hojjat et al. [46] in their study addressing salinity conditions, applied a 60% AgNP concentration and 20% silica concentration with controlled treatment of distilled water to fenugreek seeds. In their case, the seeds started to germinate after as soon as four days. The silver nanoparticles were found to improve germination capacity indicators and seed tolerance to salinity. Thus, according to these authors, the enhancement of and improvement in seed germination capacity may be expected upon seed treatment with 30 μg mL^−1^ of AgNPs. A study conducted under salt stress conditions by Sable et al. [47] showed that the application of AgNPs promoted lentil seedling growth and seed germination. As reported by Khan et al. [48], the treatment of seeds ripening in rice with photosynthesized AgNPs in concentrations of 5 and 10 ppm significantly increased seedling vigor and germination compared to the conventional hydroconditioning. In turn, Sabir et al. [49] demonstrated significant increases in root dry weight, root fresh weight, and root elongation upon seed treatment with 100 ppm AgNPs. In a study with AgNPs synthesized from *Alnus nitida* leaves and applied to wheat seeds in various solutions (0.75 μg mL^−1^, 1.5 μg mL^−1^, 3 μg mL^−1^, 6 μg mL^−1^, and 15 μg mL^−1^) under in vitro conditions, a significant increase was recorded in both dry and fresh weight in wheat seeds treated with 6 μg mL^−1^ AgNPs [48]. Antioxidant potential measured in vitro cannot be tantamount to the nutritional value without in vivo or dietary data. In the case of CuNPs, there are many reports pointing to their toxicity, including to their adverse effect on the germination capacity of seeds and growth of crops of various species, i.e., lettuce (*Lactuca sativa*), alfalfa (*Medicago sativa*), wheat (*Triticum aestivum*), mung bean (*Vigna radiate*), common bean (*Phaseolus vulgaris*), or corn (*Zea mays*) [49,50,51,52]. The negative outcomes of their application included a diminished germination capacity, decreased biomass, shortening of root and stem length, as well as modification of the photosynthesis course. Furthermore, Zafar et al. [53] concluded that CuO nanoparticles had adverse effects on black mustard seed germination and seedling growth, as Cu oxide (CuO) is the prevailing form of Cu found in the rhizosphere. In turn, Sakouhi et al. [54], who applied pure copper (Cu) in the substratum for sunflower cultivation, found a ca. 30% increase in the dry weight of leaves and stems and also improved plant vigor; however, Cu application in the same concentration contributed to 15% root growth inhibition compared to the control variant.

The application of CuONP onto rice seeds grown on a filter paper [55], in soil [56], and in the hydroponic system [57] inhibited seed germination and increased their main organs. In contrast, germination in the presence of CuONPs and the growth of rice seedling stems were not inhibited in the study by Yang et al. [58]; however, these authors observed a significant reduction in root length compared to the control variant. According to Zuverza-Mena et al. [59], the application of nano-CuO, micro-CuO, and ionic Cu diminished germination of coriander (*Coriandrum sativum*) seeds by over 50% and affected their nutritional value.

In our research, we used an innovative combination of two methods (PEF and soaking with metal nanoparticles) to check the response of sunflower seeds, manifested in their germination ability. Its results demonstrate that the coupled use of these two methods significantly affected the germination capacity of sunflower seeds as well as the stem and root length of sunflower seedlings (Table 1). The application of only the PEF method contributed to the increase in the values of the above parameters (by 0.77 and 3.63%, respectively).

### 3.2. Color Properties and Contents of Pigments

The color of sunflower seedlings is one of the characteristics that influence consumers’ choice and acceptance of the final product and is therefore considered an important factor in determining its quality. According to Wojdyło et al. [60], the contents of chlorophylls and carotenoids in sprouts of plants are lower than in their microgreens. In their study, the chlorophyll content of sprouts ranged from 6.0 to 108.5 µg g^−1^, with the highest value determined in lentil sprouts. These authors concluded that chlorophyll *a* is the primary photosynthetic pigment and that chlorophyll *b* is not indispensable for photosynthesis to occur, and, therefore, not present in all cells that perform photosynthesis. An increase in the content of chlorophyll *b* results from adaption to shade, as it allows a plant to absorb a broader range of light wavelengths, and also plays an important role in enhancing cellular metabolism [61]. A study conducted by Pandey et al. [45], who soaked Indian mustard seeds in AgNP and AgNO_3_ solutions of 100, 500, and 1000 mg L^−1^, showed that the presence of AgNP increased the content of chlorophylls at all concentrations tested, compared to the control. According to Wang et al. [62], the rate of photosynthesis depends on the chlorophyll content of plants, with its reduced content negatively affecting process rate. In turn, Tighe-Neira et al. [63] analyzed CuNPs’ accumulation in leaves of *Oryza sativa* L., *Elodea densa*, and *Lindoltia punctata* and concluded that the nanoparticles could contribute to the modification of stomata and chloroplasts. In addition, the application of CuNPs inhibited photosynthesis in spring barley and *Quercus robur* [64], whereas in the study by Ahmed et al. [65], the use of a 2000 µg L^−1^ CuONP solution contributed to tomato cell toxicity, inhibiting their growth. In turn, Tan et al. [66] observed a decrease in the total chlorophyll content in the seeds of wheat upon their treatment with a 500 mg kg^−1^ CuONP solution, which, in contrast, did not compromise the positive NPs’ effect on wheat photosynthesis. In another study with duckweed (*Lemma*), Tan et al. [66] recorded a decrease in the total content of carotenoids.

In our research the results indicate that the combined use of these two methods significantly affected the contents of chlorophyll *a*, chlorophyll *b*, and carotenoid pigments (Table 3), increasing them in sunflower seedlings.

### 3.3. Antioxidative Potential and Possible Molecular Mechanisms

Seed germination may affect the ferric ion reducing capacity, also referred to as ferric reducing antioxidant power (FRAP), content of polyphenols, content of phenolic compounds, and antioxidative activity against ABTS and DPPH radicals. In the study conducted by Pointner et al. [67], significant changes were observed during germination in the antioxidative activity and TPC. The TPC of germinating flaxseed and sunflower seeds reached 572.8 ± 13.1 mg kg^−1^ and 139.5 ± 15.2 mg kg^−1^, respectively, which may be indicative of their increased antioxidative potential and, thus, their higher oxidative stability and longer shelf-life. Zhang et al. [68] demonstrated an increased TPC in flaxseed sprouts as soon as 5 days after germination. The results reported by these authors were 236.30 ± 0.46 mg GAE kg^−1^ for TPC, 605.62 ± 0.39 μmol TE kg^−1^ for the antioxidative activity against the DPPH radical, and 1864.41 ± 4.84 μmol TE kg^−1^ for the ABTS scavenging activity, and were higher than those determined for sunflower seedlings. In turn, Wojdyło et al. [60] demonstrated that seedlings of selected plants had a higher total polyphenol content than microgreens of these plants, and reported TPC values from 26.7 to 191.1 mg 100^−1^ g m.c. in especially sprouts of radish, lentil, broccoli, and sunflower.

The activity of antioxidants (TPC) determined in sunflower seedlings is determined by various factors. Antioxidative defense may be influenced by ultraviolet-B radiation (UV-B) absorbed by sunflower leaves. According to Guo et al. [6], the synthesis and conversion of phenolic compounds proceeds via a few important molecular signaling pathways, including the pentose phenothiazide, acetate/malonate, phenylpropanoid, and shikimate oxidizing, and hydrolysable tannin pathways, and glycolysis. The total phenolic content increases after 5 days of germination. In turn, the TPC of sunflower seeds ranges from 2700 to 3611 mg 100 g^−1^ DM [69,70]. In the study by Paśko et al. [71], the germination process was found to affect the contents of total phenolics and their soluble and bound fractions not only in seeds but especially in sprouts. The total content of phenols determined in sunflower seeds was reported to increase from 1.06 to 3.60 mg g^−1^ [6].

A study conducted by Pająk et al. [9] with sprouts of various plants demonstrated an increase in their antioxidative activity determined with the ABTS assay compared to seeds, with the highest activity noted for sunflower sprouts and the lowest one for mung bean sprouts.

In our research, we also reported an increase in the antioxidative activity during seed germination in the reaction with the free DPPH radical. In this case, the highest antioxidative potential was demonstrated for sunflower seeds followed by radish, broccoli, and mung bean.

According to Guo et al. [6], the DPPH radical scavenging activity increases throughout the germination period, likely due to an increased total content of phenols, melatonin, and total isoflavones. The same observation was made by Pająk et al. [9] for sprouts of other plant species, e.g., a 12-fold increase in the case of mung bean sprouts and a 2-fold increase in the case of radish sprouts.

The combined use of a PEF and seed coating with CuNPs and AgNPs had a positive effect on the total oxidative activity of both FRAP and polyphenols in the analyzed sunflower seedlings (Table 4). The most beneficial energy level and solution of nanometals in the FRAP assay turned out to be 1 kJ·kg^−1^ and Ag50 mL·L^−1^, as it yielded the greatest increase of 125.91% in the FRAP value compared to the control variant with 1 kJ·kg^−1^ and 127.49% compared with the crude control without PEF treatment. In the case of polyphenols, the greatest increase was noted after the use of CuNPs at 25 and 50 mL·L^−1^ with 5.5 kJ·kg^−1^ and 1 kJ·kg^−1^ (by 11.64 and 10.92% compared with the corresponding control and by 15.24 and 14.40% compared to the crude control without PEF treatment).

## 4. Materials and Methods

### 4.1. Material

The experimental material included seeds of sunflower “Helosum SU” cv., being an ancient cultivar characterized by a high yielding potential. The seeds were purchased from Saatbau Polska Sp. z o.o., and grown under laboratory conditions on Petri dishes.

### 4.2. Silver and Copper Nanoparticles

The experiment was conducted with two samples of colloidal nanoparticles, being a type of suspension of particles with sizes not exceeding 100 nm suspended in a solvent. The selected nanoparticles of silver and copper with a concentration of 4000 ppm were purchased at ITP-System SP z o.o. in Dąbrowa Górnicza, Poland. They are commonly available as additives to products based on polar solvents suitable for use as plant protection agents. The quality of the purchased nanocolloids was checked by analyzing collected samples of nanoparticles under a TEM electron microscope.

### 4.3. Sample Imaging via Transmission Electron Microscopy

Samples of silver and copper were ground in an agate mortar to a fine powder, which was then mixed with 99.8% ethanol (POCH), and the suspension formed was placed in an ultrasound homogenizer for 10 s. Afterwards, a sample was collected from the suspension using a pipette and put onto copper mesh (200 mesh/inch) covered with lacey formvar stabilized with carbon (Ted Pella) and left on filter paper till the ethanol had evaporated. Then, the samples were placed in a special holder and transferred to the electron microscope. They were imaged by means of a Titan G2 60–300 kV transmission electron microscope (FEI), equipped in a field emission gun (FEG), at an electron beam acceleration voltage of 300 kV. The TEM imaging of the sample microstructure was performed in the bright field mode using a CCD camera as a detector. In this mode, imaging occurs with electrons from the zero-beam passing through the sample undiffracted.

### 4.4. Preparation of Seeds for Analyses

First stage. Sunflower seeds were placed in a 70% ethanol solution for 15 s for surface sterilization. Next, they were transferred to a 1.5% solution of sodium hypochlorite (NaClO) for 10 min, and afterwards, rinsed five times with distilled water.

Second stage. The rinsed sunflower seeds were soaked in tap water (control sample) and in tap water with the addition of silver and copper nanoparticles for 15 min in order to achieve optimal conductivity. The total soaking time was 25 min. The seeds were weighed on an AS 110.R2 analytical scale (Radwag-Radom in Poland) with a max. weight of 110 g. The weighed material was transferred to a glass vessel (250 g of seeds), which was filled with 750 mL of water. Two solutions were used with a nanoparticle content of 25 and 50 mL in 1 L of tap water. Thus, prepared samples were poured into a measuring cuvette and exposed to the electric current under the following conditions: voltage of 24 kV, intensity of 1 kV cm^−1^, 24 cm distance between electrodes, frequency of 20 Hz, and impulse width of 7 μs, in an ELEA Pulsed Electric Field system. The values of the above parameters were the same in all experimental variants. The only changing variable was the energy applied (kJ kg^−1^), i.e., 1.0 and 5.5 kJ kg^−1^. There were three control samples: I—crude control, which included seeds soaked only in water, II—control sample, which included seeds soaked in water and exposed to PEF energy of 1 kJ kg^−1^, and III—control sample, which included seeds soaked in water and exposed to PEF energy of 5.5 kJ kg^−1^.

The seeds were germinated under laboratory conditions at room temperature (20 ± 2 °C). They were sown in triplicate on Petri dishes with a diameter of 90 cm lined with three layers of filter paper, 100 seeds each, and watered with distilled water, 1.5 mL per each dish. Seven days after seed sowing, the counting of normally developed seedlings was started to determine their germination capacity, and the length of the stems and roots of the seedlings was measured.

Computations were made according to the formula for germination capacity calculation:

Germination capacity (%) = (Number of germinated seeds/Total number of seeds) × 100(1)

### 4.5. Drying Method

The freeze-drying process was carried out using an ALPHA 1–4 freeze-dryer (Martin Christ, Osterode am Harz, Germany), which operates based on a contact method of heat supply, and consists of: a drying chamber, a heating plate power supply system, a water vapor freezing system, and a control and measurement system with an interface. The dryer was equipped with a WPT 5 scale integrated with a computer, which enabled continuous monitoring of the mass of the dried material. The samples were freeze-dried at a temperature of 40 °C and a pressure of 52 Pa in the chamber.

### 4.6. Color Measurement

The color measurement was performed using the reflectance method using an X-Rite 8200 sphere spectrophotometer with a measuring aperture 12.7 mm in diameter, with the D_65_ light source and a standard colorimetric observer with a 10° field of view. Prior to each measurement, the device was calibrated using a white standard. Color measurements were performed in five replicates, for the dried material ground to a fraction size of <100 μm.

Color coordinates were determined in the CIEL*a*b* system. Color measurement in this system consists in numeric determination of the three coordinates: L*, a*, and b*, where L* denotes color brightness and fits within the range of 0 for an ideally black body to 100 for an ideally white body; a* indicates the color shift from green (−a*) to red (+a*), whereas b* denotes the color shift from blue (−b*) to yellow (+b*).

The determined color coordinates enabled determining the values of the color chromaticity (*c*) and color hue (*h*) of the dried material in the cylindrical coordinate system according to Mohammadi et al. [72].

(2) $$c=\sqrt{{\left(a*\right)}^{2}+{\left(b*\right)}^{2}}$$

(3) $${h=\tan}^{-1}\frac{b*}{a*}$$

### 4.7. Antiradical Activity

The ability to neutralize free ABTS (2,2′-azinobis (3-ethylbenzothiazoline-6-sulfonic acid)) radicals was determined using the method developed by Re et al. [73]. In turn, the ability to scavenge free DPPH (2,2-diphenyl-1picrylhydrazyl) radicals was analyzed acc. to Brand-Williams et al. [74]. The absorbance decrease was quantified on a spectrophotometer at the wavelength of 734 nm for ABTS and 517 nm for DPPH. The ability to scavenge free ABTS and DPPH radicals was expressed as EC_50_, indicating the concentration of dry matter (mg mL^−1^) inducing a 50% reduction in the initial concentration of ABTS or DPPH radicals.

### 4.8. The Content of Total Carotenoids and Chlorophylls

The contents of chlorophyll *a*, chlorophyll *b*, and total carotenoids were determined with the spectrophotometric method by means of a Hewlett-Packard 8453 Diode Array single-beam absorption spectrophotometer, operating in a 190–1100 nm wavelength range, according to Lichtenthaler [75]. This method consists of pigment extraction with an 80% acetone solution followed by reading out absorbance values at wavelengths typical of chlorophyll *a*, chlorophyll *b*, and carotenoids. To this end, an appropriate portion of the material was weighed, transferred to a porcelain mortar, mixed with 3 mL of 80% acetone, and ground for 2 min. The resulting suspension was centrifuged in a centrifuge at 15,000× *g* (g = 9.81 m s^−2^) for 3 min. The supernatant was collected quantitatively and its volume was measured. Next, 100 μL of the supernatant was collected with a pipette and mixed with 2 mL of an 80% anhydrous acetone. Then, the absorption spectrum was measured by reading out absorbance values at wavelengths typical of chlorophyll *a*, chlorophyll *b*, and carotenoids, reaching: 470 nm, 646.8 nm, and 663.2 nm, respectively. Measurements of absorption spectra were performed using a quartz cuvette (Sigma, Bandai, Japan). The contents of chlorophyll *a*, chlorophyll *b*, and total carotenoids were computed using the following formulas:

(4) $$C_{a}=12.25\times A_{663.2}-2.79\times A_{646.8}$$

(5) $$C_{b}=21.50\times A_{646.8}-5.10\times A_{663.2}$$

(6) $$C_{x+c}=\frac{1000\times A_{470}-1.82\times C_{a}-85.02\times C_{b}}{198}$$

### 4.9. FRAP Analysis

The FRAP assay followed a procedure developed by Benzie and Strain [76], and modified by Thaipong et al. [77]. To this, 50 μL of the methanolic extract of the analyzed sample was added to 1450 μL of a solution prepared from acetate buffer (pH = 3.6), 10 mmol L^−1^ of a TPTZ solution, and 20 mmol L^−1^ of an FeCl_3_⋅6H_2_O solution (mixed in a 10:1:1 ratio, *v*/*v*/*v*) and mixed thoroughly. After 8 min, absorbance was measured at λ = 593 nm using a UV 2600i plus spectrophotometer (Shimadzu, Kyoto, Japan). A blank sample contained 50 μL of redistilled water instead of the extracts. The results are expressed as Trolox equivalents (mg TE g^−1^ DM).

### 4.10. Polyphenols

To prepare extracts, 10 g portions of the material were weighed and soaked in 100 mL of a 99.8% methanol solution. Extraction was performed in a WL-1 shaker (Biosan, Riga, Latvia), at room temperature, for 60 min. The methanolic extracts obtained were stored in a fridge at a temperature of ca. 4 °C.

The total content of polyphenols was determined following the method developed by Singleton and Rossi [78], with small modifications. In brief, 0.05 mL of the extract was collected into a 25 mL measuring flask and mixed with subsequently added 2 mL of methanol (p.a., 99.8%), 10 mL of distilled water, and 2 mL of the Folin–Ciocalteu reagent (at 1:5) ratio. The mixture was left to stand for 3 min. Then, 1 mL of a 10% sodium carbonate (Na_2_CO_3_) solution was added to the mixture, which was mixed again and left for 30 min. Afterwards, the flasks were filled up with distilled water. Absorbance was measured at a wavelength of 750 nm, against the blank sample. The total polyphenol concentration is expressed in gallic acid (GA) equivalents (mg GAE g^−1^ DM).

### 4.11. Statistical Analysis

The study results were analyzed using Statistica 13 software from StatSoft. Shapiro–Wilk (normality) and Levene (homogeneity of variance) tests were performed, in accordance with the requirements for one-way analysis of variance. One-way analysis of variance (ANOVA) was performed in order to determine the significance of the impact that the respective factors had on the analyzed values. The significance of differences between the mean values was established with Tukey test. The adopted significance level was α = 0.05. All the tests and analyses were conducted in 4 replications.

## 5. Conclusions

This study confirms the viability of combining innovative techniques affecting the germination capacity and chemical composition of sunflower seedlings, i.e., PEF and seed coating with metal nanoparticles. Its results demonstrate that the coupled use of these two methods significantly affected the germination capacity of sunflower seeds as well as the stem and root length of sunflower seedlings. The high PEF energy coupled with a high solution of nanoparticles (CuNPs in particular) may induce oxidative stress resulting in the inhibition of germination but, at the same time, activates antioxidative pathways that enhance the biological potential of seedlings as functional foods.

The study findings underscore the potential use of PEF and nanoparticle-based seed treatment as a tool for modulating antioxidant levels in edible seedlings. However, due to the observed suppression of germination under specific conditions, the optimization of NP solutions and PEF intensity is essential prior to implementing such treatments in seed technology or food production.

## Figures and Tables

**Table 1:** Effect of the solution of metal nanoparticles and pulsed electric field treatment on the germination capacity of sunflower seeds and on the stem length and root length of sunflower seedlings.

Sample	PEF Energy Unit	Germination Capacity	Stem Length	Root Length
		mm		
crude control	kJ kg^−1^	91.00 ± 0.01 ^a^	70.00 ± 1.12 ^a^	59.00 ± 1.50 ^a^
control-1		91.70 ± 0.58 ^aa^	70.70 ± 1.15 ^aa^	60.30 ± 1.53 ^ab^
control-5.5		94.30 ± 1.15 ^ab^	57.70 ± 1.53 ^ab^	76.30 ± 1.52 ^ab^
Cu25-1		96.30 ± 0.58 ^bb^	52.30 ± 2.08 ^bb^	61.00 ± 1.73 ^ab^
Cu50-1		89.70 ± 1.53 ^bb^	45.70 ± 1.15 ^bb^	59.00 ± 0.90 ^ba^
Cu25-5.5		71.70 ± 0.59 ^bb^	61.70 ± 1.53 ^bb^	45.00 ± 1.60 ^bb^
Cu50-5.5		91.00 ± 1.00 ^ba^	60.30 ± 1.58 ^bb^	76.70 ± 1.53 ^ab^
Ag25-1		96.30 ± 1.53 ^bb^	86.70 ± 1.53 ^bb^	80.70 ± 2.08 ^bb^
Ag50-1		93.30 ± 2.52 ^bb^	78.70 ± 1.50 ^bb^	79.70 ± 1.53 ^bb^
Ag25-5.5		52.70 ± 0.58 ^ab^	37.70 ± 1.53 ^bb^	70.70 ± 2.09 ^bb^
Ag50-5.5		90.70 ± 1.15 ^ba^	84.70 ± 1.48 ^bb^	79.70 ± 1.54 ^bb^

^a,b^—the first letter in each column means the comparison with the appropriate control after using PEF; the second letter in each column means the comparison with the first crude control (*p* < 0.05); the same letter as in the control means no significant differences.

**Table 2:** Color analysis of sunflower seedlings.

Sample	PEF Energy Unit	*L*	*a*	*b*	*C*	*h*
crude control	kJ kg^−1^	50.15 ± 0.39 ^a^	−1.28 ± 0.63 ^a^	21.48 ± 0.44 ^a^	21.52 ± 0.48 ^a^	86.63 ± 1.6 ^a^
control 1		48.78 ± 0.31 ^ab^	−2.11 ± 0.19 ^aa^	25.83 ± 0.55 ^ab^	25.92 ± 0.55 ^ab^	85.34 ± 0.41 ^ab^
control 5.5		53.8 ± 0.37 ^ab^	−1.88 ± 0.40 ^aa^	25.22 ± 0.14 ^ab^	25.29 ± 0.17 ^ab^	85.74 ± 0.88 ^aa^
Cu25-1		47.70 ± 0.17 ^bc^	−3.38 ± 0.18 ^bc^	24.76 ± 0.28 ^bb^	24.99 ± 0.28 ^ab^	82.22 ± 0.42 ^bc^
Cu50-1		52.82 ± 0.14 ^bc^	−2.94 ± 0.30 ^bb^	25.52 ± 0.33 ^bc^	25.69 ± 0.34 ^ab^	83.44 ± 0.65 ^bc^
Cu25-5.5		44.68 ± 0.25 ^bc^	−4.25 ± 0.23 ^bc^	27.34 ± 0.27 ^bc^	27.67 ± 0.30 ^bb^	81.18 ± 0.41 ^bb^
Cu50-5.5		51.16 ± 0.25 ^bc^	−1.74 ± 0.32 ^aa^	22.31 ± 0.32 ^ba^	22.38 ± 0.33 ^bb^	85.55 ± 0.81 ^ab^
AG25-1		53.21 ± 0.3 ^bc^	−1.74 ± 0.37 ^aa^	22.73 ± 0.54 ^ba^	22.8 ± 0.56 ^ba^	85.63 ± 0.84 ^aa^
Ag50-1		48.00 ± 0.42 ^ab^	−1.44 ± 0.24 ^aa^	22.97 ± 0.24 ^ba^	23.01 ± 0.25 ^ab^	86.42 ± 0.57 ^ba^
Ag25-5.5		53.29 ± 0.48 ^ab^	−3.96 ± 0.14 ^bc^	27.56 ± 0.36 ^bb^	27.85 ± 0.35 ^bc^	81.82 ± 0.33 ^bc^
Ag50-5.5		55.09 ± 0.32 ^ab^	−1.59 ± 0.51 ^ab^	23.72 ± 0.38 ^bb^	23.78 ± 0.40 ^bc^	86.19 ± 1.18 ^ab^

^a,b,c^—the first letter in each column means the comparison with the appropriate control after using PEF, the second letter in each column means the comparison with the first crude control (*p* < 0.05); and the same letter as in the control means no significant differences.

**Table 3:** Contents of chlorophylls and carotenoids in sunflower seedlings.

Sample	PEF Energy Unit	Chlorophyll *a*	Chlorophyll *b*	∑ Carotenoids
		(mg 100 g^−1^ DM)		
crude control	kJ kg^−1^	36.4 ± 0.46 ^a^	19.2 ± 0.15 ^a^	34.6 ± 0.29 ^a^
control-1		36.7 ± 0.24 ^aa^	18.5 ± 0.11 ^aa^	34.1 ± 0.42 ^aa^
control-5.5		36.8 ± 0.61 ^aa^	17.8 ± 0.13 ^ab^	35.3 ± 0.28 ^ab^
Cu25-1		38.4 ± 0.4 ^bb^	17.9 ± 0.09 ^ab^	36.6 ± 0.29 ^ab^
Cu50-1		38.4 ± 0.47 ^bb^	19.8 ± 0.08 ^ba^	36.2 ± 0.39 ^ab^
Cu25-5.5		38.7 ± 0.43 ^bb^	16.4 ± 0.11 ^bc^	37.0 ± 0.53 ^bc^
Cu50-5.5		38.3 ± 0.38 ^bb^	20.6 ± 0.11 ^bc^	36.3 ± 0.36 ^bb^
AG25-1		38.2 ± 0.41 ^bc^	21.3 ± 0.05 ^b^	37.5 ± 0.36 ^bb^
Ag50-1		38.9 ± 0.33 ^bc^	20.7 ± 0.15 ^bb^	37.7 ± 0.49 ^bc^
Ag25-5.5		40.2 ± 0.15 ^bc^	22.8 ± 0.11 ^bb^	38.3 ± 0.29 ^cb^
Ag50-5.5		41.6 ± 0.22 ^bc^	22.2 ± 0.05 ^bc^	39.4 ± 0.20 ^cb^

^a,b,c^—the first letter in each column means the comparison with the appropriate control after using PEF; the second letter in each column means the comparison with the first crude control (*p* < 0.05); and the same letter as in the control means no significant differences.

**Table 4:** Antioxidative potential of sunflower seedlings.

Sample	PEF Energy Unit	FRAP	Polyphenols	TPC	ABTS	DPPH
		[mg TE g^−1^ DM]	[mgGAE g^−1^ DW]		[μmol Trolox g^−1^ DW]	
crude control	kJ kg^−1^	10.20 ± 0.03 ^a^	42.23 ± 0.06 ^a^	21.10 ± 0.22 ^a^	78.20 ± 0.70 ^a^	46.70 ± 0.09 ^a^
control-1		11.08 ± 0.04 ^aa^	43.59 ± 0.04 ^ab^	20.20 ± 0.13 ^aa^	79.60 ± 0.93 ^aa^	46.10 ± 0.25 ^aa^
control-5.5		10.91 ± 0.03 ^aa^	44.27 ± 0.04 ^ab^	20.70 ± 0.31 ^aa^	80.60 ± 0.28 ^ab^	48.30 ± 0.31 ^ab^
Cu25-1		12.85 ± 0.02 ^bc^	48.67 ± 0.09 ^bc^	21.60 ± 0.14 ^ab^	82.20 ± 0.54 ^bc^	48.80 ± 0.29 ^bc^
Cu50-1		12.18 ± 0.04 ^bc^	48.35 ± 0.02 ^bc^	21.70 ± 0.16 ^ba^	81.50 ± 0.65 ^bc^	48.40 ± 0.32 ^bc^
Cu25-5.5		12.95 ± 0.03 ^bc^	49.19 ± 0.04 ^bc^	22.60 ± 0.11 ^bc^	80.00 ± 0.23 ^ab^	49.80 ± 0.32 ^bc^
Cu50-5.5		12.34 ± 0.03 ^bc^	48.88 ± 0.05 ^bc^	21.50 ± 0.24 ^ba^	82.70 ± 0.23 ^bc^	49.30 ± 0.16 ^bb^
AG25-1		12.18 ± 0.04 ^bc^	45.53 ± 0.04 ^bc^	20.80 ± 0.05 ^aa^	80.60 ± 0.47 ^ab^	50.40 ± 0.11 ^bc^
Ag50-1		12.05 ± 0.03 ^ac^	45.31 ± 0.03 ^bc^	22.70 ± 0.28 ^bc^	82.60 ± 0.63 ^bc^	48.90 ± 0.21 ^bc^
Ag25-5.5		11.53 ± 0.05 ^ab^	47.96 ± 0.02 ^bc^	22.90 ± 0.21 ^bc^	84.70 ± 0.32 ^bc^	51.40 ± 0.30 ^bc^
Ag50-5.5		11.31 ± 0.02 ^ab^	46.19 ± 0.08 ^bc^	23.90 ± 0.13 ^bc^	82.00 ± 0.38 ^bc^	51.20 ± 0.29 ^bc^

^a,b,c^—the first letter in each column means the comparison with the appropriate control after using PEF; the second letter in each column means the comparison with the first crude control (*p* < 0.05); the same letter as in the control means no significant differences.

## Data Availability

The original contributions presented in this study are included in the article. Further inquiries can be directed to the corresponding author.
